# Decreased Plasma Aβ in Hyperlipidemic APP_SL_ Transgenic Mice Is Associated with BBB Dysfunction

**DOI:** 10.3389/fnins.2016.00232

**Published:** 2016-06-01

**Authors:** Tina Löffler, Stefanie Flunkert, Magdalena Temmel, Birgit Hutter-Paier

**Affiliations:** Neuropharmacology, QPS AustriaGrambach, Austria

**Keywords:** Alzheimer's disease, high-fat diet, cholesterol, APP mice, plasma Aβ, blood brain barrier (BBB), cerebrovascular disorders

## Abstract

Besides the continued focus on Aβ and Tau in Alzheimer's disease (AD), it is increasingly evident that other pathologic characteristics, such as vascular alterations or inflammation, are associated with AD. Whether these changes are an initial cause for the onset of AD or occur as a result of the disease in late stages is still under debate. In the present study, the impact of the high-fat diet (HFD) induced vascular risk factor hyperlipidemia on Aβ levels and clearance as well as cerebral vasculature and blood-brain barrier (BBB) integrity was examined in mice. For this purpose, human APP transgenic (APP_SL_) and wildtype (WT) mice were fed a HFD for 12 weeks. Plasma and tissues were subsequently investigated for Aβ distribution and concentrations of several vascular markers. Decreased plasma Aβ together with increased levels of insoluble Aβ and amyloid plaques in the brains of HFD fed APP_SL_ mice point toward impaired Aβ clearance due to HFD. Additionally, HFD induced manifold alterations in the cerebral vasculature and BBB integrity exclusively in human APP overexpressing mice but not in wildtype mice. Therefore, HFD appears to enhance Aβ dependent vascular/BBB dysfunction in combination with an increased proportion of cerebral to plasma Aβ in APP_SL_ mice.

## Introduction

With advancing age Alzheimer's disease (AD) is the major cause of dementia. The two main histo-pathological features of AD are the extracellular deposition of amyloid β peptides (Aβ) in plaques and the formation of intracellular tangles mainly composed of hyperphosphorylated Tau protein (Selkoe, 2001). Aβ is a proteolytic product of the amyloid precursor protein (APP), generated by sequential β-secretase and γ-secretase cleavage. While in rare cases of early onset AD the disease is caused by mutations in the genes for APP or Presenilins, the majority of late-onset AD (LOAD) patients do not have a mutation in one of the genes (Tanzi and Bertram, 2005; Bell and Zlokovic, 2009). Therefore, cerebral accumulation of Aβ in LOAD patients is supposed to be a result of an imbalance between increased production and decreased clearance of Aβ by so far unknown reasons (Hardy and Selkoe, 2002). The current understanding implies that Aβ is released from the brain across the blood-brain barrier (BBB) into the blood (Podlisny et al., 1990), mainly via low density lipoprotein receptor-related protein 1 (LRP1)-mediated clearance (Kang et al., 2000; Shibata et al., 2000; Bates et al., 2009). Additionally, a converse mechanism, leading to an influx of Aβ into the brain exists that is mediated by the receptor for advanced glycation end products (RAGE; Mackic et al., 1998; Deane et al., 2003). Generally, the integrity of the BBB is of high importance for maintaining healthy brain function, since it prevents a passive exchange of solutes between the blood and the brain. During aging and in AD, several alterations in the cellular elements of the BBB can be observed, including: loosening of tight junctions, accumulation of extracellular matrix components in the vascular basement membrane, decreased endothelial mitochondrial density and changes in the expression of endothelial receptors/transporters (Marques et al., 2013). Additionally, several other cerebrovascular abnormalities have been identified in AD brains such as increased microvascular density, increase of inflammatory markers, changes in vessel diameter, atherosclerotic plaques and/or cerebral amyloid angiopathy (CAA; Farkas and Luiten, 2001). It is still unclear, whether these changes are the initial cause for the onset of AD or occur as a result of the disease. In the last years, increasing evidence suggests that hypercholesterolemia and other vascular risk factors may contribute to the pathogenesis of LOAD (Skoog et al., 1999; Humpel, 2011).

Similar to human AD tissue, various of these cerebrovascular abnormalities like CAAs, changes in cerebral angiogenesis or BBB integrity, were observed in different APP transgenic mouse models (van Dooren et al., 2005; Biron et al., 2011). In the here presented study we investigated the additional impact of hyperlipidemia on BBB integrity as well as Aβ levels and clearance in human APP transgenic (APP_SL_) and wildtype (WT) mice. Animals received a high-fat diet (HFD) or standard diet (ND) for 12 weeks starting at an age of 3 months. Harvested tissues were subsequently analyzed for Aβ levels and distribution, as well as for several vascular and BBB markers.

HFD seems to impact cerebral Aβ clearance observed as decreased plasma Aβ with concomitantly increased levels of insoluble Aβ and plaques in the brain of APP_SL_ mice. Additionally, HFD induced several changes in the cerebral vasculature and BBB integrity in mice overexpressing mutated human APP.

## Materials and methods

### Animals

APP_SL_ transgenic mice (Havas et al., 2011; Löffler et al., 2014), overexpressing human APP with Swedish and London mutation by the mThy1 promoter, and non-transgenic littermates (WT) were housed in individually ventilated cages under a constant light-cycle (12 h light/dark). Room temperature and humidity were kept constant at approximately 24°C and 40–70%, respectively. Transgenic and WT animals of both sexes were fed either a high-fat diet (HFD – D12451 mod, see Table 1) or standard rodent chow (ND, both ©Ssniff, Soest, Germany) starting at the age of 3 months and pursuing the diet for 12 weeks. During this period, animals' weights were recorded once weekly to follow weight changes. Chow and normal tap water were available *ad libitum*. Animal studies conformed to the Austrian guidelines for the care and use of laboratory animals and were approved by the legal authorities.

**Table 1:** **Diet composition of the high fat diet (HFD) in comparison to the normal diet (ND)**.

**Crude nutrients (%)**	**HFD**	**ND**	**Fatty acids (%)**	**HFD**	**ND**
Dry matter	93.9	94.8	C 14:0	0.29	0.02
Crude protein	22.5	17.8	C 16:0	5.15	0.55
Crude fat	23.1	5.1	C 16:1	0.62	0.03
Crude fiber	5.7	5.0	C 18:0	2.83	0.24
Crude ash	5.9	5.3	C 18:1	8.99	1.33
N free extracts	36.8	61.9	C 18:2	3.19	2.65
Starch	6.6	36.8	C 18:3	0.37	0.32
Sugar	20.4	14.8	C 20:0	0.01	0.02
Dextrin	11.1	11.0	Cholesterol (mg/kg)	175	−

### Tissue sampling

To be able to obtain plasma of fasted mice, chow was removed from the cages 4–5 h before tissue sampling. From each tested mouse, blood and brain tissue were sampled after sedation. Blood was collected into heparin-coated vials and consequently used to obtain plasma (1000 × g, 10 min at RT). The collection of brain tissue was performed as described in Löffler et al. (2014). Hippocampi and cortices of the left brain hemispheres were used for biochemical analyses. The right hemispheres of all mice were fixed by immersion in freshly produced 4% paraformaldehyde/PBS (pH 7.4) at room temperature for 1 h, followed by 24 h incubation in 15% sucrose/phosphate buffered saline solution for cryo-conservation. Frozen hemispheres were stored at −80°C until further histological processing.

### Plasma lipid measurements

Total cholesterol was determined by Fluitest CHOL, high density lipoprotein (HDL) by Fluitest HDL direct and low density lipoprotein (LDL)-cholesterol, by Fluitest LDL direct assay (all Analyticon Biotechnologies, Lichtenfels, Germany). Plasma of all animals was diluted 1:2 in 0.2% NaCl solution and assays were carried out in accordance with the supplied manuals.

### Homogenization of frozen tissue samples

Frozen tissue samples were weighed and Tissue Homogenization Buffer (THB; 250 mmol/L sucrose, 1 mmol/L ethylenediaminetetraacetic acid (EDTA), 1 mmol/L ethylene glycol tetraacetic acid (EGTA), 20 mmol/L Tris, pH 7.4) including 1 × protease inhibitor cocktail (Calbiochem, Darmstadt, Germany) was added. For cortex samples, 1 mL THB per 100 mg tissue was added and for hippocampal samples, 3 mL THB was used for 100 mg tissue and homogenized with the Tissue Ruptor at greatest speed (Qiagen, Düsseldorf, Germany).

### Extraction of non-plaque associated proteins (soluble Aβ)

For extraction of non-plaque associated proteins, 100 μl of the THB homogenate were mixed with 100 μl diethylamine (DEA) solution (0.4% DEA, 100 mM NaCl). The mixture was centrifuged for 1 h at 74,200 × g, 4°C. 170 μl of the supernatant were transferred to a 1.5 ml reaction tube and neutralized with 17 μl 0.5 M Tris, pH 6.8.

### Extraction of deposited proteins (insoluble Aβ)

For extraction of deposited proteins, 100 μl of the THB homogenate were mixed with 220 μl cold formic acid (FA) and sonicated for 1 min on ice. 300 μl of this solution were transferred to a centrifugation tube and centrifuged for 1 h at 74,200 × g, 4°C. After centrifugation, 210 μl of the supernatant were transferred to a fresh tube and mixed with 4 ml FA Neutralization Solution (1 M Tris, 0.5 M Na_2_HPO_4_, 0.05% NaN_3_).

### Measurement of Aβ in cortical and plasma samples

Plasma and cortical samples (DEA and FA fraction) of all groups were analyzed for Aβ_1−38_, Aβ_1−40_ and Aβ_1−42_ with MSD® 96-well MULTI-SPOT® 4G8 Abeta Triplex Assay (Mesoscale Discovery, Rockville, USA). The immunosorbent assay was carried out according to the manual and plates were analyzed on the Sector Imager. The assay detects human as well as rodent Aβ species.

### Semi-quantitative ELISA

For all ELISA measurements, samples were diluted in carbonate-bicabonate buffer pH 9.5, 30 μl were added to each well of a NUNC Maxi-Sorp 96 well plate (Thermo Scientific, Waltham, USA) and incubated overnight at 4°C without agitation. Plates were then washed three times with 250 μl/well of ELISA-wash buffer (50 mM Tris, 0.14 M NaCl, 0.05% Tween-20, pH 8.0) and blocked for 30 min with 100 μl blocking buffer (50 mM Tris, 0.14 M NaCl, 1% BSA, pH 8.0). After blocking, plates were washed once and 40 μl of the respective primary antibody diluted in blocking buffer + 0.05% Tween-20 were added and incubated while shaking for 2 h at room temperature (RT). Plates were again washed three times and 40 μl of the corresponding horseradish-peroxidase (HRP)-conjugated secondary antibody were added. After 1 h incubation at RT on a plate shaker, plates were washed three times and 100 μl of ultrasensitive TMB substrate (Applied Biological Materials Inc., Richmond, Canada) were added. After 30 min incubation in the dark, reaction was stopped with 100 μl 0.5 M H_2_SO_4_ and measured at 450 nm with μQuant universal microplate spectrophotometer. Relative differences between groups were expressed as x-fold change to WT on normal diet (ND). To be in a linear range, optimal dilutions of antibodies and samples were tested prior to the measurements. Antibodies: anti-mouse IgG biotinylated (Eubio, BA-2000; 1:5000) + anti-streptavidin HRP conjugated (Zymed, 43-4323; 1:5000); anti-occludin (abcam, ab167161; 1:1000) + anti-rabbit IgG, HRP-linked (GE-Healthcare, NA934; 1:5000); anti-ZO1 tight junction protein (abcam, ab59720; 1:100) + anti-rabbit IgG, HRP-linked (GE-Healthcare, NA934; 1:2500); anti-Vascular Cell Adhesion Molecule 1 (VCAM-1) (Santa Cruz, sc-1504-R; 1:500) + anti-rabbit IgG, HRP-linked (GE-Healthcare, NA934; 1:5000); anti-LRP1 [alpha-2-macroglobulin receptor] (Santa Cruz, sc-57351; 1:500) + anti-mouse IgG, peroxidase linked (GE-Healthcare, NA931; 1:1000); anti-Cluster of Differentiation 31 (CD31) (abcam, ab28364; 1:250) + anti-rabbit IgG, HRP-linked (GE-Healthcare, NA934; 1:1000).

### Immunoblotting

Equal amounts of protein were separated on a 10% SDS-polyacrylamide gel and transferred onto a 0.45 μm nitrocellulose membrane using a wet blot chamber (Bio-Rad, Hercules, USA). The blots were incubated overnight with the primary antibody at 4°C. Proteins were detected using Western-Bright ECL spray (Advansta, Menlo Park, USA) after incubation for 1 h at room temperature with the appropriate secondary antibody. Primary antibodies used: anti-ZO1 tight junction protein (abcam, ab59720; 1:100); anti-Vascular Cell Adhesion Molecule 1 (VCAM-1) (Santa Cruz, sc-1504-R; 1:500). Secondary antibody: anti-rabbit IgG, HRP-linked (GE-Healthcare, NA934; 1:5000).

### Immunofluorescence

From every group 4 animals were randomly chosen for histological examination. Cryo-conserved brains were embedded in O.C.T. tissue freezing medium (Leica biosystems, Nussloch, Germany) and cut sagittally from the medial to the lateral side. The brain was divided into 12 layers, each containing 30 slices á 10 μm thick, according to the mouse brain atlas of Paxinos & Franklin 2nd edition. Five 10-μm-thick mounted sections deriving from five different medio-lateral levels (L2, 4, 6, 8, and 11) per animal were labeled using specific antibodies. In brief, for quantification of plaque load, sections were labeled with 6E10 antibody (Signet, 9320-02, 1:1000), detected with a Cy3 conjugated goat anti-mouse antibody (Jackson, 115-165-166, 1:500). The boundaries of the entire hippocampus and cerebral cortex gray matter were delineated and measured and immunoreactive area within each region was quantified above threshold and a minimal size of 7 μm^2^ using rater-independent automated image analysis software (Image Pro Plus, version 6.2) relative to the respective region size. For quantification of vascular density and vascular Aβ, sections were co-stained with antibodies detecting collagen IV (anti-collagen IV, abcam, ab6586, 1:700) and Aβ40/42 (anti-Aβ protein MOAB2, Biosensis, M-1586-100, 1:1000) and detected with anti-rabbit IgG conjugated with Alexa Fluor 555 (abcam, ab150066, 1:500) and anti-mouse IgG conjugated with DyLight 650 (abcam, ab98797, 1:500), respectively. Again, areas of interest were the entire cerebral cortex and hippocampus gray matter. Labeling was then quantified by threshold-based detection. For quantification of exclusively vascular Aβ, collagen IV was measured using a fill holes option and a counting bi-level mask was saved. The inverted mask was mathematically subtracted from the Aβ40/42 channel to allow a separate Aβ detection on exclusively collagen IV-positive vasculature.

## Results

### Implication of HFD on body weight and plasma cholesterol level

**Figure 1:**
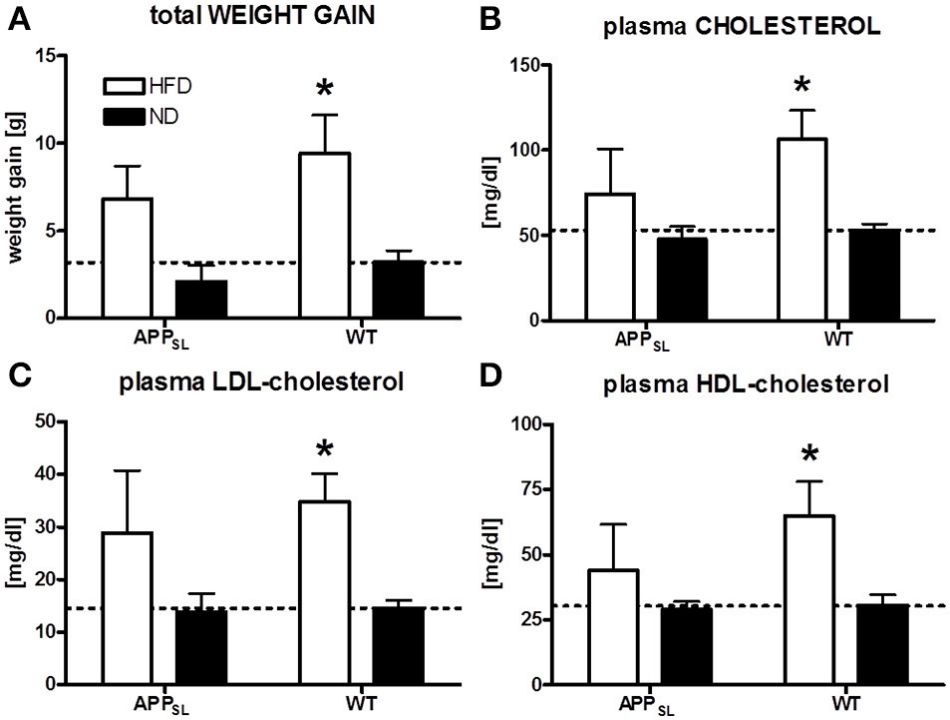
**Impact of HFD on weight gain and plasma cholesterol levels**. Comparison of final weight gain due to different diets in APP_SL_ and WT mice **(A)**. Total cholesterol **(B)**, LDL- **(C)**, and HDL-cholesterol **(D)** levels in the plasma of HFD and ND fed animals of both genotypes. *N* = 5–8 animals per group. Statistical analyses: Two-way-ANOVA with Bonferroni's post-test, ^*^p < 0.05.

WT mice responded to HFD feeding with a significant increase in weight gain and plasma total cholesterol, HDL- and LDL-cholesterol levels (Figure 1) compared to WT mice on ND. In APP_SL_ transgenic animals, also a tendency to increased weight gain and higher cholesterol levels on HFD was observed, but lacking significance (Figure 1).

### Changes in the amount and distribution of cerebral and plasma Aβ levels

**Figure 2:**
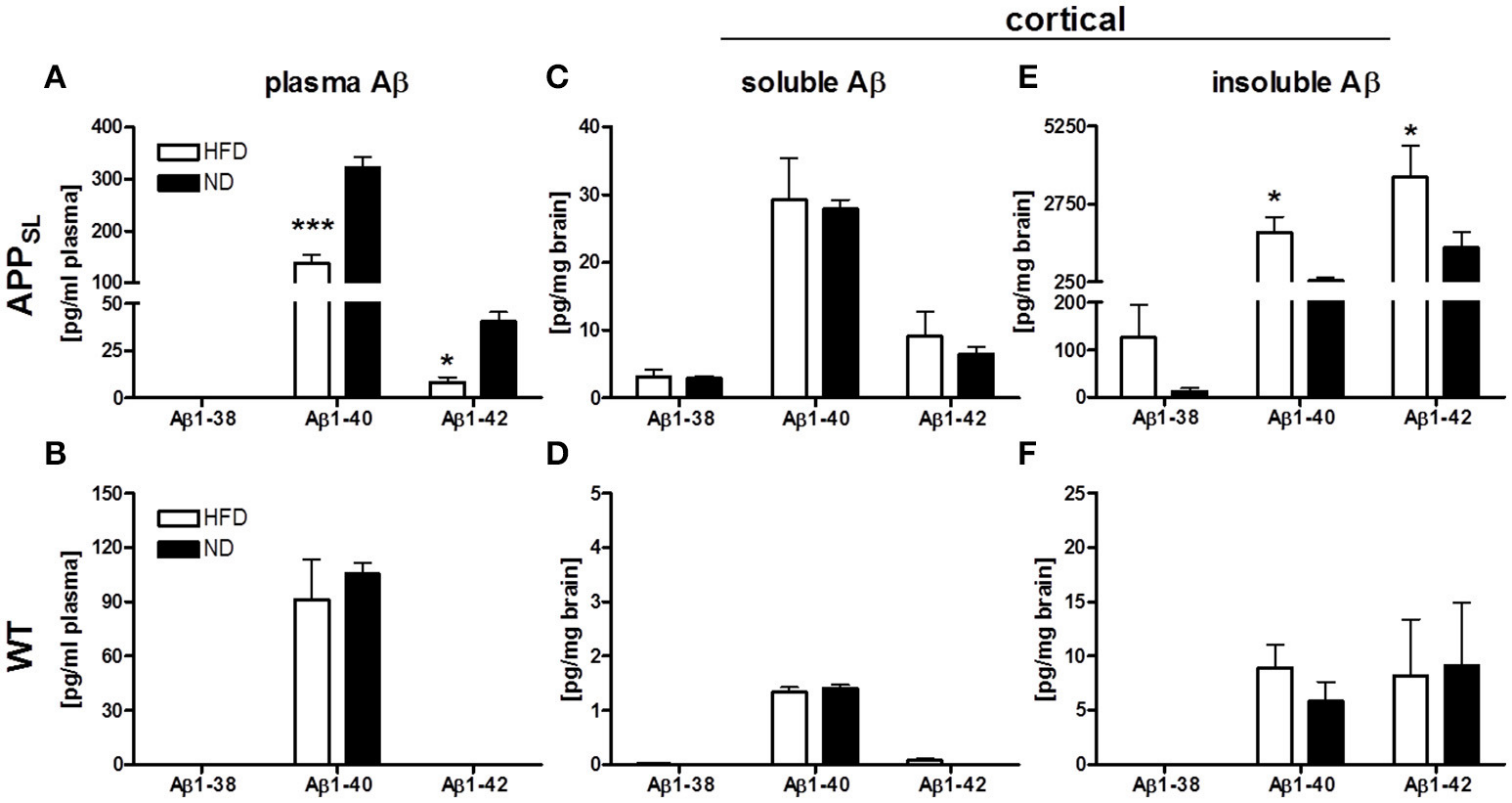
**Impact of HFD on Aβ levels in plasma and cortex of APP_**SL**_ and WT mice**. Levels of Aβ_1−38_, Aβ_1−40_ and Aβ_1−42_ in the plasma of APP_SL_ **(A)** and WT **(B)** mice on HFD and ND. Cortical soluble Aβ species on HFD and ND in APP_SL_ **(C)** and WT **(D)** mice as well as levels of cortical insoluble Aβ species on HFD and ND in APP_SL_ **(E)** and WT **(F)** mice. *N* = 5–8 animals per group. Statistical analyses: Two-way-ANOVA with Bonferroni's post-test, ^*^p < 0.05, ^***^ *p* < 0.001.

Three Aβ species, Aβ_1−38_, Aβ_1−40_, and Aβ_1−42_, were measured in the plasma and cortex of all animals on both diets using an immunosorbent assay which detects human and rodent Aβ. Due to overexpression of mutated human APP, all Aβ species were found to be profoundly increased in APP_SL_ mice compared to WT animals only expressing endogenous Aβ. In the plasma, Aβ_1−40_ was the most abundant Aβ species in APP_SL_ as well as WT mice (Figures 2A,B). HFD led to a profound decrease of Aβ1-40 and Aβ1-42 levels in plasma of APP_SL_ mice at about 50 and 70%, respectively (Figure 2A). WT mice also showed a tendency toward reduced plasma Aβ1-40 levels (Figure 2B), but this effect was not significant. While in cortical soluble fractions no differences between diets were observed (Figures 2C,D), insoluble Aβ1-40 and Aβ1-42 levels were found to be significantly increased in HFD fed APP_SL_ mice compared to APP_SL_ mice on ND (Figure 2E). In WT mice, diet had no significant effect on levels of any cerebral Aβ species (Figures 2D,F).

**Figure 3:**
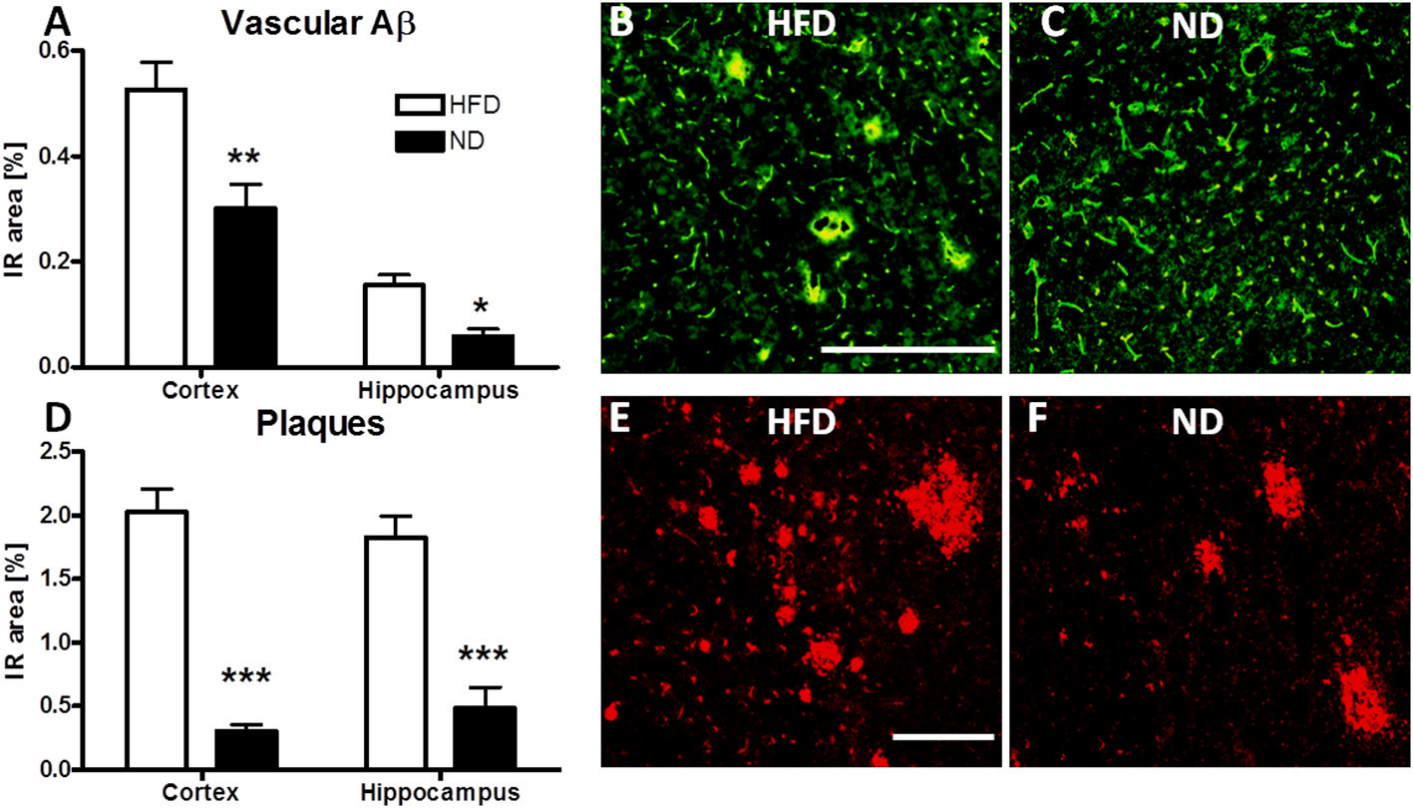
**Immunofluorescent evaluation of vascular Aβ and amyloid plaques in HFD fed APP_**SL**_ mice**. Immunoreactive area (IR) of vascular Aβ in the cortex and hippocampus of HFD and ND fed APP_SL_ mice **(A)**. Representative picture of vascular Aβ labeling in the cortex of a HFD fed **(B)** and a ND fed **(C)** APP_SL_ mouse, illustrating the overlap (yellow) between CollagenIV (green) and MOAB2 labeled Aβ (red). Scale bar: 100 μM. IR area of plaques in cortex and hippocampus of HFD and ND fed APP_SL_ mice **(D)**. Representative picture of plaque load in the cortex of a HFD fed **(E)** and a ND fed **(F)** APP_SL_ mouse, labeled with 6E10 antibody. Scale bar: 100 μm. *N* = 4 animals per group. Statistical analyses: Two-way-ANOVA with Bonferroni's post-test, ^*^p < 0.05, ^**^p < 0.01, ^***^p < 0.001.

To further define the allocation of cerebral Aβ in APP_SL_ mice, immunofluorescent evaluation of vascular Aβ and plaque load was carried out. HFD led to a highly significant increase in both, Aβ at the cerebral microvasculature as well as plaque load in the cortex and hippocampus of APP_SL_ transgenic mice (Figure 3).

### Alterations of vascular integrity due to genotype and/or HFD

**Figure 4:**
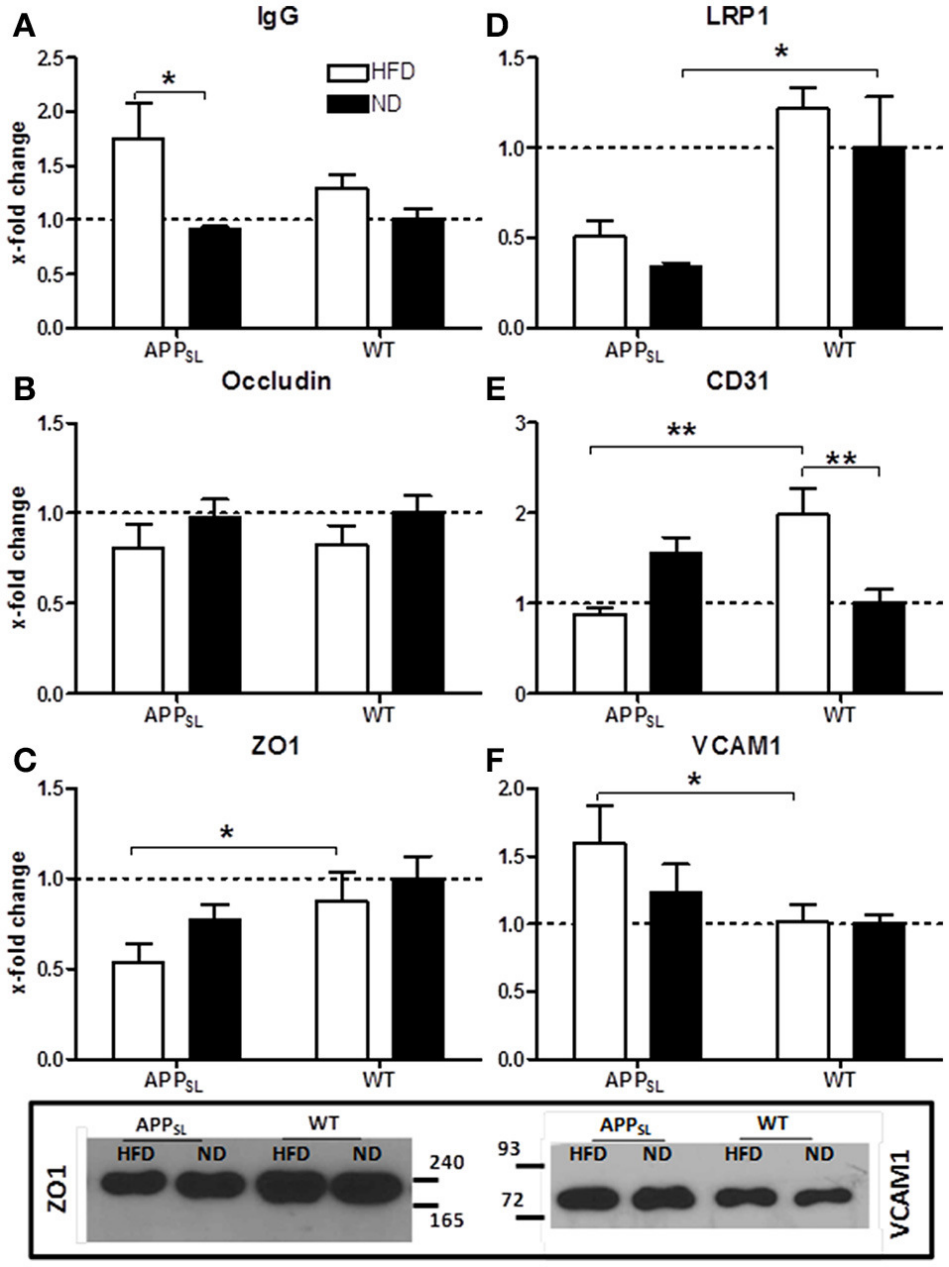
**Changes in protein levels of EC and BBB integrity associated markers due to HFD and genotype measured with ELISA**. IgG influx into the cortex in APP_SL_ and WT mice on HFD and ND **(A)**. Cortical levels of the tight junction markers Occludin **(B)** and ZO1 **(C)** in APP_SL_ and WT mice on both diets. LRP1 **(D)** as well as EC marker CD31 **(E)** and VCAM1 **(F)** levels in the cortex of APP_SL_ and WT mice on ND and HFD. Relative differences between groups were expressed as x-fold change to WT on ND. *N* = 5–8 animals per group; Statistical analyses: Two-way-ANOVA with Bonferroni's post-test, ^*^p < 0.05, ^**^p < 0.01. Additional western blots of ZO1 and VCAM1.

To investigate the permeability of the BBB and the condition of tight junctions, protein levels of total IgG, occludin and Zona Occludens 1 (ZO1) in cortices of all animals were determined. HFD led to a significantly increased influx of IgG into the cortex of APP_SL_ mice compared to ND fed APP_SL_ mice, while in WT mice only a tendency to higher IgG influx due to HFD was observed (Figure 4A). Occludin levels were only slightly decreased due to HFD in both, APP_SL_ and WT mice (Figure 4B). In contrast, levels of ZO1 seemed to be rather dependent on the genotype than on the diet. Already on ND, ZO1 showed a tendency to be reduced in APP_SL_ mice compared to WT on ND (Figure 4C). This difference turned significant when comparing APP_SL_ mice on HFD with WT mice on HFD (Figure 4C). Expression of the low density lipoprotein receptor-related protein 1 (LRP1) that is involved in receptor-mediated flux of Aβ across the BBB, was also found to be mainly dependent on the genotype (Figure 4D). Already on ND, levels of LRP1 were found to be significantly decreased in APP_SL_ mice compared to WT control mice (Figure 4D). Levels of CD31, a general marker for endothelial cells and angiogenesis, showed a quite different profile. While on ND levels of CD31 even showed a tendency to be increased in APP_SL_ compared to WT mice, this picture changed completely on HFD (Figure 4E). In WT mice, HFD induced a significant increase in CD31 levels compared to ND leading to a highly significant difference in CD31 levels between WT mice on HFD and APP_SL_ mice on HFD (Figure 4E). Levels of Vascular Cell Adhesion Molecule 1 (VCAM1) were also investigated as a marker for activated ECs and inflammatory processes and were found to be significantly increased in APP_SL_ mice on HFD compared to WT mice on HFD (Figure 4F).

## Discussion

In the last years, more and more evidence accumulates that AD and vascular changes are linked (Pallebage-Gamarallage et al., 2010). Multiple cerebrovascular abnormalities have been identified in AD brains and clinical studies indicate that individuals with vascular risk factors, like increased plasma cholesterol, are more susceptible to AD (Puglielli et al., 2003). Therefore, several studies were carried out investigating the impact of different diets on APP metabolism, plaque load and cognition in rodents (Refolo et al., 2000; Oksman et al., 2006; Perez et al., 2010). The common outcome of these studies suggests, that dietary alterations act on APP processing and plaque load, either beneficial when administering high doses of unsaturated fatty acids (Perez et al., 2010), or worsening when treating animals with e.g., high-fat/high-cholesterol diets (Oksman et al., 2006).

In the present study, we investigated the impact of a high-fat/high-cholesterol diet (HFD) on cerebral Aβ levels and distribution. Additionally we examined changes of BBB integrity and cerebrovascular alterations as a possible reason for the detrimental effects of HFD. Therefore, human APP transgenic (APP_SL_) and wildtype (WT) mice received a HFD or standard diet (ND) for 12 weeks, starting at an age of 3 months. WT mice responded very well to HFD feeding, with increased weight gain and plasma cholesterol levels compared to ND fed WT mice. In APP_SL_ animals the response was weaker, lacking significance in all measured weight- and cholesterol parameters. This influence of cerebral APP overexpression on peripheral (lipid-) metabolism was previously described, indicating APP as a possible sensor for increased cholesterol levels with down regulating function (Löffler et al., 2016). The current knowledge postulates that Aβ is released from the brain into the plasma across the BBB (Podlisny et al., 1990) mainly via LRP1 mediated transcytosis (Kang et al., 2000; Shibata et al., 2000). Levels of different Aβ species in the plasma have been investigated in patients as diagnostic markers of LOAD with contradictory results (Bates et al., 2009). Most studies found an overlap of plasma Aβ_1−40_ and Aβ_1−42_ levels between control and AD subjects, thus limiting its diagnostic power in human patients. In mice plasma Aβ levels are so far poorly investigated (Van Dorpe et al., 2000; Kandimalla et al., 2005; Galloway et al., 2007). Interestingly, in the present study plasma Aβ_1−40_ and Aβ_1−42_ were found to be exceedingly decreased due to HFD feeding in APP_SL_ animals. This effect could be due to a “lipid-masking effect,” since Aβ was shown to be associated with lipoproteins in the plasma (Galloway et al., 2007; Mamo et al., 2008); but in APP_SL_ mice on ND the highly increased plasma Aβ levels can be assumed to be brain derived. Due to the sole neuronal expression of human APP in these mice, Aβ found in the plasma supposably passed the BBB. Decreased plasma Aβ levels on HFD therefore point toward a possible clearance problem across the BBB. Together with the high increase of all insoluble Aβ species in the brain of HFD fed APP_SL_ mice, impaired clearance of Aβ from the brain due to HFD feeding can be assumed. By examining the cerebral distribution of Aβ via immunohistochemical methods, significantly increased plaque formation was detected. This is in line with the here presented finding of increased insoluble Aβ species and can also be associated with increased levels of BACE1 mRNA as previously described (Löffler et al., 2016). Additionally, a high proportion of Aβ co-localized with the cerebral vasculature was found in HFD fed APP_SL_ mice, suggesting that Aβ accumulates in and around vessel walls. These results also indicate impaired clearance mechanisms or increased Aβ influx at the BBB due to HFD. Shibata et al. (2000) first demonstrated that LRP1 is mainly responsible for the clearance of Aβ_1−40_ across the BBB. The authors also found a reduction of LRP1 levels in human AD brains, particularly in regions associated with extensive Aβ deposition. In APP_SL_ mice LRP1 levels were also found to be significantly reduced compared to WT littermates, but the HFD had no additional influence on LRP1 expression.

Markers associated with BBB integrity appeared to be influenced by HFD and genotype. Tight junctions between endothelial cells (ECs) in brain capillaries are the most important structural elements of the BBB. BBB permeability has already been previously described in different APP transgenic mouse lines (Poduslo et al., 2001). In the present study, the additional effect of HFD on BBB markers in APP transgenic vs. WT mice became apparent. Especially the abundance of the junction-associated protein Zona Occludens 1 (ZO1) was shown to be significantly reduced in APP_SL_ mice on HFD compared to WT mice on HFD. Reduction or re-localization of the occludin-ZO1 complex from tight junctions is contributing to an increase in paracellular permeability (Rao et al., 2002) which could also be detected in APP_SL_ mice on HFD observed as increased influx of IgG into the cortex. That decreased Aβ efflux and increased BBB permeability are not contradictory, but rather interrelated, was already shown in human AD patients and *in vitro* studies (Gonzalez-Velasquez et al., 2008; Hartz et al., 2012; Marques et al., 2013). Additionally, inflammatory processes also seem to be involved in EC dysfunction and the breakdown of the BBB (Marques et al., 2013; Grammas et al., 2014). In the present study, an increase in VCAM1 expression, a marker for activated ECs, was detected in APP_SL_ mice due to HFD. The VCAM1 protein is known to mediate the adhesion of lymphocytes and monocytes to ECs and therefore may also be involved in BBB disruption (Eibl and Benoit, 2004; Priglinger et al., 2004). All these results indicate a general slight impairment of BBB function in APP_SL_ mice, getting highly significant when fed a HFD.

Angiogenesis seems to be differently regulated in APP_SL_ and WT mice on the two applied diets. While HFD led to a significant increase in CD31 levels in WT mice, in APP_SL_ mice a decrease of this angiogenesis marker was observed. This phenomenon of increased angiogenesis due to HFD in WT mice was already previously described (Yi et al., 2012). In contrast, reduction of angiogenesis in HFD fed APP_SL_ mice further completes the picture of highly compromised cerebral vasculature in these animals. Since almost all of the investigated BBB and vascular markers were stronger affected in HFD fed APP_SL_ mice than in corresponding WT mice the influence of APP overexpression on these parameters has to be emphasized. Although HFD was the triggering factor for the observed severe changes in vascular/BBB integrity, most effects appeared only in the presence of increased APP/Aβ levels in APP_SL_ mice. Therefore, the often described detrimental effects of Aβ on cerebral vasculature (Park et al., 2004; Hartz et al., 2012) seem to be enhanced by HFD feeding in the present study.

Since in WT mice on HFD only marginal changes in BBB integrity could be detected, the assumption that changes in the cerebral vasculature due to HFD feeding lead to an impaired Aβ clearance and enhanced plaque formation should be revised. We now hypothesize that in APP over-expressing mice HFD feeding enhances Aβ-dependent vascular/BBB impairment, leading to reduced Aβ clearance and hence to a higher proportion of plaques as well as of Aβ accumulation on and around cerebral vessels.

## Author contributions

TL designed, performed, and interpreted the experiments and wrote the manuscript. SF edited the manuscript. MT performed parts of histological experiments and edited the manuscript. BH conceived the study, designed and interpreted experiments and edited the manuscript.

### Conflict of interest statement

The authors declare that the research was conducted in the absence of any commercial or financial relationships that could be construed as a potential conflict of interest.

## Abbreviations

AD: Alzheimer's disease
Aβ: amyloid beta
APP: amyloid precursor protein
BBB: blood-brain-barrier
CAA: cerebral amyloid angiopathy
HDL: high density lipoprotein
HFD: high-fat diet
LDL: low density lipoprotein
LOAD: late-onset AD
LRP1: LDL-receptor-related protein 1
RAGE: receptor for advanced glycation end products
VCAM1: Vascular Cell Adhesion Molecule 1
ZO1: Zona Occludens 1.
